# Home Enzyme Replacement Therapy in Gaucher Disease: A Review

**DOI:** 10.3390/jcm14030842

**Published:** 2025-01-27

**Authors:** Beata Kieć-Wilk, Paul Guijt, Michaela Dan, Magy Abdelwahab, Shoshana Revel-Vilk, Christine Serratrice

**Affiliations:** 1 Unit of Rare Metabolic Diseases, Department of Pathophysiology, Jagiellonian University Medical College, 31-121 Krakow, Poland; mbkiec@gmail.com; 2 Metabolic Disease Clinic, The St. John Paul II Specialist Hospital, 31-202 Krakow, Poland; 3 International Gaucher Alliance, London EC2A 4NE, UK; paul.guijt@outlook.com; 4 Romanian Gaucher Association, 400540 Cluj-Napoca, Romania; michaela.dan@neuf.fr; 5 Cairo University Pediatric Hospital Pediatric Hematology and BMT Department and Gaucher Disease Clinic, Kasr Elainy Hospital, Cairo 11562, Egypt; magywahab@yahoo.com; 6 Gaucher Unit, The Eisenberg R&D Authority, Shaare Zedek Medical Center, Jerusalem 9103102, Israel; srevelvilk@gmail.com; 7 Faculty of Medicine, Hebrew University, Jerusalem 9103102, Israel; 8 Division of Internal Medicine for the Aged, University Hospital of Geneva, Thônex, 1256 Geneva, Switzerland; 9 School of Medicine, University of Geneva, 1206 Geneva, Switzerland

**Keywords:** Gaucher disease, home therapy, enzyme replacement therapy, safety, compliance

## Abstract

Since the early 1990s, Gaucher Disease has been a pioneering condition for home-based enzyme replacement therapy (ERT), marking a significant shift in patient care. Since then, many countries have adopted this approach. However, home ERT is not possible in all countries. **Objectives:** The aim of this article is to explore the implementation of home ERT for Gaucher disease, focusing on patient expectations, safety, compliance, economic benefits, and practical considerations. **Methods:** The PRISMA reporting protocol was followed, focusing on articles about home ERT for Gaucher Disease. **Results:** Twenty articles were analyzed in the review, revealing promising outcomes. Home ERT has consistently been shown to be safe, to improve patients’ quality of life, to reduce the utilization of hospital resources, and to pose no compliance issues. **Conclusions:** We believe it is essential to expand the availability of home ERT for Gaucher Disease to all countries where ERT is accessible. Based on the literature review, we present the conditions that must be met before starting home ERT programs.

## 1. Introduction

Enzyme replacement therapy (ERT) has been available for patients with Gaucher disease (GD) since the early 1990s [1]. ERT can be administered in hospitals or at home based on patient needs, resource availability, and safety considerations. Hospital administration ensures medical supervision, which is vital for managing potential adverse reactions but is costlier and less convenient. Home therapy, while more comfortable and cost-effective, demands well-trained patients or caregivers and robust support systems, potentially reducing hospital resource strain but increasing the need for monitoring infrastructure, impacting the National Healthcare System through resource reallocation and potential cost efficiencies. In some countries, home infusion, including self-infusion, was advocated from the outset. Currently, in many countries, patients have the option to receive ERT at home. However, there are still countries or regions where home ERT is not approved or not reimbursed [2].

The COVID-19 pandemic, which impacted the delivery of ERT in hospitals worldwide, prompted the International Gaucher Alliance (IGA), an organization bringing together all organizations of patients with GD, to investigate with its global community the current provision of home ERT and how it is organized in each country. Their project aimed to facilitate a proper home ERT provision, especially in countries where home ERT is unavailable, and to develop guidelines and potentially other informative resources. The International Working Group on Gaucher Disease (IWGGD) aims to promote clinical and basic research into GD and strives to improve the quality of life for patients. Bringing together GD experts, healthcare professionals, and patients in an open forum for discussion, the IWGGD members are working on consensus guidelines for treating patients with GD (https://www.iwggd.com). The IGA and IWGGD have joined forces to expand the use of home ERT in regions where it is not yet available. In this article, we present a literature review following PRISMA guidelines [3] on home ERT for GD and provide recommendations for the necessary steps and conditions to be met before implementing such a program.

## 2. Materials and Methods

**Figure 1:**
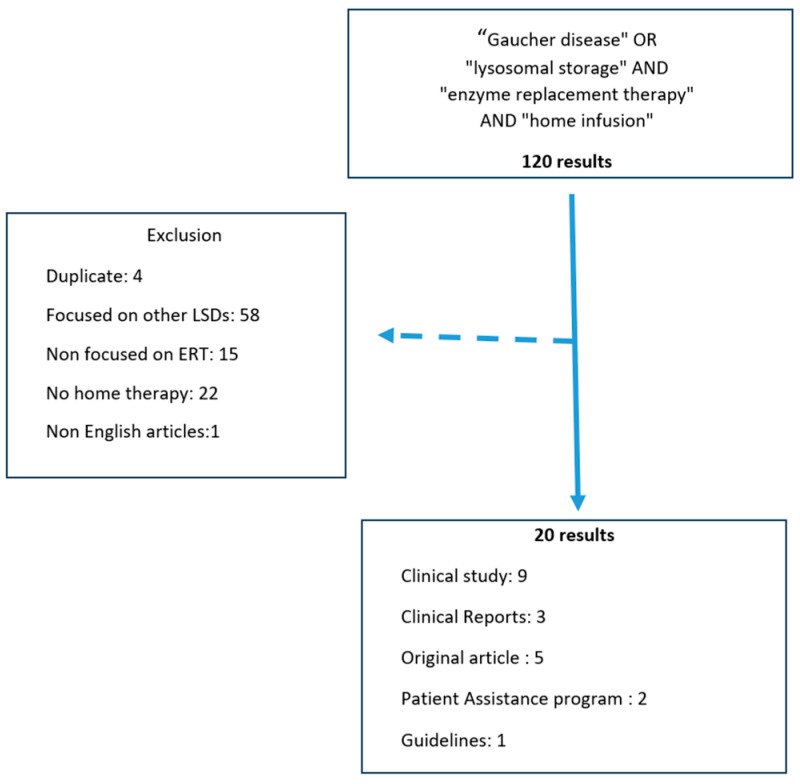
Flow chart of literature search [2,4,5,6,7,8,9,10,11,12,13,14,15,16,17,18,19,20,21,22].

A search of the literature was conducted in Pubmed/Medline database, Cochrane Review and Google Scholar with Windows version 0.24121.37.0, No entry date was set, and the search was revised in November 2024. The keywords used were as follows: ((“Gaucher Disease”[Mesh]) OR Gaucher Disease, OR (“lysosomal storage”[Mesh])) OR lysosomal storage AND (“enzyme replacement therapy”[Mesh]) OR Enzyme replacement Therapy AND “Home therapy” [Mesh] OR Home therapy to extract original studies, expert opinions, guidelines, and reports published in international journals. This investigation was conducted by two assessors. One hundred twenty results were retrieved. Extracted publications were screened for relevance and duplication. Studies discussing patients with LSDs other than type 1 GD, non-enzymatic home therapy, or non-English articles were excluded. Twenty relevant articles were then analyzed (Figure 1).

## 3. Results

### 3.1. Historical Overview

GD was the first lysosomal storage disease (LSD) to be treated with ERT [1]. ERT has been shown to be highly effective in treating hematological, visceral, and bone manifestations of GD [23]. It is a lifelong treatment administered via intravenous infusion every two weeks. GD was also the first LSD to be treated with home ERT [16]. In the first international collaborative study, the authors reported that home ERT for GD was safe, feasible, and well accepted by the patients and their families [16]. The primary motivation for opting for home ERT was to reduce the burden related to recurrent hospital visits. Since then, clinical trials, including those involving children, have confirmed the high safety profile of home ERT [4,5,6,7].

### 3.2. Patients Perspectives

In a survey published by Milligan et al. [9] comparing hospital therapy versus home ERT in the United Kingdom (UK), the authors found that 21 out of 25 adult patients with type 1 GD preferred home ERT. These patients initially received ERT in the hospital for at least a year before transitioning to home administration. Interestingly, although most patients preferred home ERT, half of the patients reported that being treated in a hospital was not an added burden. The main stressors associated with hospital-based ERT were the need to travel to the hospital, the hospital environment itself, waiting for the treatment to be administered, and missing work or school days. However, some patients expressed concerns about the quality of health monitoring while undergoing home ERT. Patients who preferred in-hospital treatment cited a perceived increased safety, as well as the opportunity for interaction and exchange with other patients, as the greatest benefits. Conversely, most patients reported that home ERT did not negatively impact their family life. According to the survey results, most patients with type 1 GD felt that home ERT was “more effective” and easier to accept. The authors emphasized that patients adjusted rapidly to receiving therapy at home. Only 2 out of 25 patients opted to continue receiving in-hospital ERT, finding home ERT more stressful than hospital therapy.

### 3.3. Safety of Home ERT

While home ERT significantly improves patient comfort and quality of life, the question of safety is crucial. Although ERT for GD is generally well tolerated, ensuring safety in a home setting is essential.

The first report on home ERT for GD was an international collaborative study published in 1993, focusing on alglucerase home infusion [16]. The authors reported the safety and feasibility of low-dose/high-frequency home ERT in 33 patients with GD. They also highlighted the feasibility of a venous access device implanted into 24 patients to address vein access issues and the necessity of regular visits by the nurse.

Subsequent publications showed that home ERT was safe for both adults and children with type 1 GD, based on both clinical trials and real-life experience [6]. In another study, 18 out of 35 patients reported no problems with home ERT [10]. Those who did experience issues reported only minor adverse events (AEs) such as fever while still feeling more in control of their treatment and condition.

Home ERT was also offered as an option for velaglucerase alfa in the four open-label clinical studies [11] to eligible patients who received their initial ERT in the hospital (Table 1). A total of 104 patients receiving at least one infusion of velaglucerase alfa at home were included in these four trials [7]. No safety concerns related to the location of the treatment (home versus hospital) were observed. All patients underwent their initial infusions in the hospital before transitioning to home care.

In another study, Zimran et al. evaluated the safety of progressively decreased infusion time of velaglucerase alfa [17], allowing for home ERT in the final phase. Fifteen patients were included, and the infusion was accelerated gradually from 60 min to 10 min without any adverse events (AEs), and the return to home settings was also uneventful. This possibility was corroborated by two additional studies from the same team [5,22] (Table 1).

A safety analysis that included all taliglucerase alfa AEs reported in a global safety database retrieved data for 163 cases [4]; among them, 33 were associated with home use (19 definite, 14 possible). None were fatal, 14 were serious, and 19 were non-serious. No specific or increased risk associated with home administration was noted (Table 1).

### 3.4. Experience During the COVID-19 Pandemic

In recent years, the SARS-CoV-2 pandemic has highlighted some limitations of the global healthcare system. It necessitated changes in the organization of work, shifting certain procedures outside of hospital settings. New challenges arose in managing care for patients with chronic diseases. In a 2021 report, 44.6% of 92 healthcare professionals treating patients with LSD, including GD, mentioned that access to ERT was a critical issue during the COVID-19 pandemic [24]. Access was not only impacted by hospital ERT protocols but also by self-isolation or care restrictions due to COVID-19 infection or fear of infection [25]. A panel of experts published recommendations related to the COVID-19 pandemic and GD treatment, emphasizing that ERT must be continued without prolonged interruption. While missing one or two infusions is generally not harmful for most patients, a prolonged interruption must be avoided. Home ERT was proposed as a solution to avoid prolonged interruptions, particularly during crisis situations [19,20].

### 3.5. Home ERT and Compliance

While home ERT provides greater independence for patients, treatment compliance can be a concern for healthcare providers. In a study by Hughes et al. involving patients with type 1 GD, more than half have received home imiglucerase for over 6 years. Among these, 21 out of 35 reported that they had never missed a dose [10]. For those who did miss doses, it was typically due to vacations lasting longer than two weeks. Additionally, nearly all patients considered themselves to be highly cooperative with their healthcare team. Clinical studies have shown that home ERT can improve patient compliance. In a clinical trial with velaglucerase alfa, the challenge of long distances traveled for hospital-based infusions was highlighted [18]. Home ERT was introduced to address this issue, and its effectiveness was reflected by the inclusion of home treatment in the protocol after the extension study began. Recently, Revel-Vilk et al. reported excellent (≥95%) annual compliance with taliglucerase alfa at home [4].

### 3.6. Medical Requirements/Resources to Start Home ERT

Home ERT must be implemented with a focus on safety. According to Hughes et al. [10], one hospital-based infusion is generally sufficient for adult patients with type 1 GD. However, in practice, patients often receive three infusions in a hospital setting before transitioning to home ERT [4,21]. In some countries, like the Netherlands, the first infusion can take place at home under the supervision of a trained nurse (Carla Hollak’s personal communication). Home ERT requires a well-organized and regulated community infrastructure, individual assessments of patient suitability, and protocols for the management of possible infusion-related reactions. A small number of patients may still prefer in-hospital ERT, often due to hospital proximity, challenges with venous access, or the need for other specialist procedures and consultations that cannot be performed at home [10]. Respecting patient choice is essential.

Although self-infusion is sometimes practiced in some countries, we did not find any articles evaluating this practice in GD.

### 3.7. Economic Advantages

Home-based ERT offers additional financial benefits for the healthcare system, including reduced use of hospital resources such as treatment rooms and nursing staff. The significant financial savings have made home ERT a favorable option for third-party payers (funding agencies depending on the country: Ministry of Health, insurance companies, etc.) [26].

A recent budget impact assessment from the perspective of the United States (US) payers found that home ERT costs 25% to 50% less than ERT administered in outpatient infusion clinics or hospitals [26].

## 4. Discussion

According to the literature, all ERTs registered for GD (imiglucerase, velaglucerase alfa, and taliglucérase according to FDA/EMA approvals) can be administered at home [4,7,27]. This review found that home ERT in GD was safe and well-accepted by the patients, who found it to be more comfortable and less stressful, with improvements in their quality of life. It also reduced hospitalization costs, and there was no issue with compliance. Enzyme replacement therapy for patients with GD was the first to have been administered at home. Thanks to this initial successful experience, patients with Fabry disease (FD) were also home treated [28,29]. As with GD, home ERT was associated with an improvement in the quality of life, enhancing patients’ ability to manage their own care and increasing their independence, particularly for those who learned to self-cannulate. Receiving home ERT and, possibly, learning how to administer the ERT independently could mitigate the negative impact caused by frequent hospital visits and associated travel [29]. Due to the positive and encouraging results, the implementation of ERT at home was then started for other LSDs such as mucopolysaccharidosis (MPS) type I, MPS type II and VI, and Pompe disease [30,31,32,33]. To date, more than 15 ERTs are home-administered.

Most of the patients with GD prefer to be treated at home. This preference is also reflected in surveys of LSDs in general. In two surveys conducted in Italy, not specifically focused on GD, the authors reported the negative impact on the quality of life when ERT is administered in hospitals [8]. The first survey was a nationwide study involving reference centers. It revealed that only a small percentage of patients (2.6%) received ERT at home; the majority were treated in a local hospital or at reference centers. The procedures of the Italian health service do not permit home ERT in every region. The second survey was a regional study in Lombardy, where 12% of patients received ERT at home. The results clearly indicated that in-hospital ERT significantly affects the quality of life of these patients.

Furthermore, pandemics highlight the crucial role of home therapy for LSD, including GD. In one Italian experience during the COVID-19 pandemic, half of the patients receiving ERT in hospitals experienced disruptions while only a few of those treated at home were affected. The primary reasons were fear of infection and the re-organization of the infusion centers [34]. According to patients, in-hospital ERT is disruptive, leading to lost days at school or work, stress, and family problems, whereas patients treated at home did not encounter these issues. Additionally, studies on home ERT emphasize increased treatment compliance and an improvement in quality of life for both patients and their families [35]. Home therapy can restore a degree of independence, allowing patients to schedule their own treatment at times that do not interfere with work commitments and family life. Moreover, clinical trials for new therapies in LSDs are conducted with a limited number of patients, and patients may refuse to participate if participation requires hospital treatment. Any participant dropping out of a trial can significantly impact its results. Therefore, the use of home ERT can enhance patients’ compliance and facilitate the collection of reliable and clinically valuable data. These various studies underscore the importance of involving the patient in the choice of treatment modalities, particularly during chronic diseases.

According to the literature, ERT for GD is usually very well tolerated, with most side effects occurring in the first three months of treatment and usually being non-severe. Although antibodies against the enzyme may develop, they are not always associated with side effects [27,36]. Most of the AEs associated with ERT in GD are type B side effects, i.e., idiosyncratic, bizarre, or novel responses that cannot be predicted by the pharmacology and are often the result of an immune reaction (allergy) to the drug [37]. In cases of an allergic reaction to an ERT, switching to a different ERT should ideally be performed in a hospital setting, with the first infusion administered under medical supervision. At home, certain precautions should be taken: patients must be trained to recognize AEs and know how to stop the infusion, possibly taking an antihistamine and/or paracetamol and/or corticosteroids. They must be able to contact their healthcare professional. It is recommended, though not mandatory, that a third person be present during the infusion. All AEs should be reported to the reference center and documented in the patient’s records. If prophylactic medication is used to prevent allergic reactions, these drugs should be included in this patient’s administration protocol before ERT administration and reviewed regularly.

Based on the literature review, we developed recommendations for home ERT in GD (Table 2). Written materials and informational leaflets should be provided to the patient regarding the organization of the home infusion.

By analogy with FD, home ERT for GD can also offer economic benefits. An interesting analysis was carried out in a study from Norway [38], modeling the resource implications of managing adults with FD. The authors noted that in an average year, patients receiving ERT for FD are expected to make 586 visits to their family practitioner’s office for ERT, which equates to 128 eight-hour days dedicated to ERT. The authors concluded that increasing the proportion of adults with FD receiving home-based ERT could free-up community-based resources, thereby improving the efficiency of medical care for other patients in the public healthcare system in Norway. Additional economic advantages include time savings, less time off work due to illness, or more time available for family responsibilities.

In some countries, ERT can be administered by self-infusion or infusion by a third party. This decision, of course, requires a discussion between the patient and the treating physician, as well as training in the technique. However, no literature on self-infusion was found for GD.

## 5. Conclusions

Home ERT offers several advantages over hospital-based therapy: it eliminates the need for frequent and regular hospital visits, provides the patient with greater independence and a sense of control over the disease, reduces the risk of exposure to potential hospital-acquired infections, and, from an economic standpoint, reduces the use of hospital resources. Home ERT can be integrated into the patient’s daily life, reducing the impact of treatment on work and family life, and thereby significantly improving the quality of life for the patient and his/her relatives. The authors recommend that, with patient agreement and after verifying the eligibility criteria, home ERT should be offered to all patients with GD, which is not yet possible in all countries where ERT is available. The IGA and the IWGGD are continuing their work together to develop guidelines for the proper, safe, cost-effective, and satisfactory implementation of home ERT for GD.

## Figures and Tables

**Table 1:** Summary of the studies dedicated to home enzyme replacement therapy for Gaucher disease.

	Alglucerase Cohort Study (Zimran et al. 1993) [16]						
	Patients (n)		Model of treatment	Patient participation in the home therapy option		Duration that patients received home therapy	
	33			100%		13 and 82 weeks per patient.	
	**Imiglucerase survey (Milligan et al. 2006) [9]**						
	Patients (n)		Survey participation	Home therapy preference		Conclusions	
	49		25	21		Home therapy is reported as more comfortable, less stressful, more effective and had less impact on family life	
**Velaglucerase alfa clinical studies (** **Elstein et al. 2017) [7]**							
Study	Number of patients	patients who received at least 10% of their infusions at home	Infusions required at clinic before home therapy	% of home infusions *		Years of home therapy **	
	n	n		Median	Range	Median y	Range
All studies	318	100		87.5	11.6–100	0.57	0.04–4.56
025EXT	10	7	71	92.1	77.3–94.8	4.41	3.88–4.56
032/039–044	57	13	23/29	87.5	11.6–97.6	2.41	0.50–3.49
034–044	40	27	4	91.2	26.4–100	2.53	0.39–3.37
058	211	53	3	73.7	18.2–100	0.33	0.04–1.04
TKT025	12						1.25–1.5
TKT025EXT	10	7 (70%)				6.75	
**Rapid infusion of Velaglucerase alfa (Zimran, 2018) [17]**							
n = 15, mean age 32 (range 22–44) years	The volume for each infusion in the study was set at 100 mL; decreased infusion time of velaglucerase alfa from 60 to 10 min using a step-wise reduction in time and allowing for home infusions in the final phase.				87%	9 months	
**Home treatment with Taliglucerase alfa (** **Revel-Vilk et al. 2023) [4]**							
Patients participating in the study	Median (range) age at the time of home therapy initiation		The annual compliance rate throughout the study period (2016–2021)	Duration that patients received home therapy n (%)			
173 patients (Israel—58, the US—61, Brazil—48, Australia—6)	38 (2–87) years		(≥95%)	12(6.9%) in the cohort received taliglucerase alfa infusions for a total of ≥7 years 47 (27.3%) in the cohort received taliglucerase alfa infusions for a total of ≥5 years 25 (14.6%) in the cohort received taliglucerase alfa infusions for a total of ≥3 years 53 (30.7%) in the cohort received taliglucerase alfa infusions for a total of ≥1 year 35 (20.4%) in the cohort received taliglucerase alfa infusions for a total of <1 year			

* In this study, median % was the median of the percentage of home infusions received by individual patients (Elstein D et al. 2017). ** Duration of home ERT in the publication was calculated as the date of a patient’s last home infusion in the study minus the date of their first home infusion. (Zimran 1993). Patients with Gaucher disease had received infusions in the hospital for a mean of 11.6 months, and home therapy for a mean of 44.6 months. In the study’s questionnaire, 3 (12%) of patients with Gaucher disease reported no symptoms, 11 (44%) reported pain, 6 (24%) reported fatigue, and 2 (8%) reported other medical symptoms (stroke and enlarged spleen). Milligan et al. (2006) [9].

**Table 2:** Conditions that must be met before starting home enzyme replacement therapy for Gaucher Disease.

Patient/caregiver agreement	The patient (or caregiver) gives informed consent for home ERT (according to local regulations)
Initial in-hospital-based therapy	Based on the experiences of practitioners and patients, it is advisable to give the first 1 to 3 infusions in the hospital. Depending on local experiences and guidelines, adjustments may be made
Clinical status	The patient is clinically stable
Safety	There are no ERT infusion-associated reactions There are no ongoing serious AEs There are no venous access difficulties (or there is a venous access device) A protocol for the management of AEs is available, and the patient/caregiver/home care team is adequately trained in the management and has access to medications for immediate treatment of infusion reactions
Logistical set-up	Close collaboration between the hospital team, patient, caregiver(s) and the medical staff administering home ERT is essential Providing a convenient and reliable system for delivering drugs and medical materials to the patient Trained nurses supporting home treatment, responsible for ERT administration (treatment is individualized to the needs of each patient, expertise in venous access device if required) The home environment should be suitable for delivery of a clinical product Patients with history of infusion reactions should have a rescue pack of medication
Infusion rate	The infusion rate should be the same as in the hospital and should not be changed in the home settings without a physician’s recommendation
GD specialist agreement	The GD specialist assesses the possibility of home infusion and signs an agreement
Primary physician availability	The primary physician will be informed of the home ERT. A physician is available, if needed, within 1 h from the patient’s home and will be available for consultation in the event of an IAR
Conditions that must be met during home ERT	
Contact with reference center	Patients and their family members must have the telephone numbers of the reference center, the emergency contacts, and the physician/nurse to contact for any AEs.

## Data Availability

All data extracted from the included studies are publicly available in PubMed (https://pubmed.ncbi.nlm.nih.gov/), in Google Scholar (https://scholar.google.com/), and Cochrane Library (https://www.cochranelibrary.com/advanced-search). The accessed date was on 15 November 2024.
